# Characterization of virus-derived small interfering RNAs in *Apple stem grooving virus*-infected in vitro-cultured *Pyrus pyrifolia* shoot tips in response to high temperature treatment

**DOI:** 10.1186/s12985-016-0625-0

**Published:** 2016-10-06

**Authors:** Juan Liu, XueJiao Zhang, YueKun Yang, Ni Hong, GuoPing Wang, Aiming Wang, LiPing Wang

**Affiliations:** 1 State Key Laboratory of Agricultural Microbiology, Wuhan, Hubei 430070 People’s Republic of China; 2 Laboratory of Key Lab of Plant Pathology of Hubei Province, Wuhan, Hubei 430070 People’s Republic of China; 3 Shihezi University, Shihezi City, Xinjiang Uyghur Autonomous Region 832003 People’s Republic of China; 4 London Research and Development Centre, Agriculture and Agri-Food Canada, London, ON N5V 4 T3 Canada

**Keywords:** *Pyrus pyrifolia*, Gene silencing, *Apple stem grooving virus*, High temperature, RT-qPCR, Virus-derived small interfering RNA (vsiRNA), Argonaute (AGO), RNA dependent RNA polymerase (RDRs), Dicer-like (DCL)

## Abstract

**Background:**

Heat treatment (known as thermotherapy) together with in vitro culture of shoot meristem tips is a commonly used technology to obtain virus-free germplasm for the effective control of virus diseases in fruit trees. RNA silencing as an antiviral defense mechanism has been implicated in this process. To understand if high temperature-mediated acceleration of the host antiviral gene silencing system in the meristem tip facilitates virus-derived small interfering RNAs (vsiRNA) accumulation to reduce the viral RNA titer in the fruit tree meristem tip cells, we used the *Apple stem grooving virus* (ASGV)–*Pyrus pyrifolia* pathosystem to explore the possible roles of vsiRNA in thermotherapy.

**Results:**

At first we determined the full-length genome sequence of the ASGV-Js2 isolate and then profiled vsiRNAs in the meristem tip of in vitro-grown pear (cv. ‘Jinshui no. 2’) shoots infected by ASGV-Js2 and cultured at 24 and 37 °C. A total of 7,495 and 7,949 small RNA reads were obtained from the tips of pear shoots cultured at 24 and 37 °C, respectively. Mapping of the vsiRNAs to the ASGV-Js2 genome revealed that they were unevenly distributed along the ASGV-Js2 genome, and that 21- and 22-nt vsiRNAs preferentially accumulated at both temperatures. The 5′-terminal nucleotides of ASGV-specific siRNAs in the tips cultured under different temperatures had a similar distribution pattern, and the nucleotide U was the most frequent. RT-qPCR analyses suggested that viral genome accumulation was drastically compromised at 37 °C compared to 24 °C, which was accompanied with the elevated levels of vsiRNAs at 37 °C. As plant Dicer-like proteins (DCLs), Argonaute proteins (AGOs), and RNA-dependent RNA polymerases (RDRs) are implicated in vsiRNA biogenesis, we also cloned the partial sequences of *PpDCL2,4*, *PpAGO1,2,4* and *PpRDR1* genes, and found their expression levels were up-regulated in the ASGV-infected pear shoots at 37 °C.

**Conclusions:**

Collectively, these results showed that upon high temperature treatment, the ASGV-infected meristem shoot tips up-regulated the expression of key genes in the RNA silencing pathway, induced the biogenesis of vsiRNAs and inhibited viral RNA accumulation. This study represents the first report on the characterization of the vsiRNA population in pear plants infected by ASGV-Js2, in response to high temperature treatment.

**Electronic supplementary material:**

The online version of this article (doi:10.1186/s12985-016-0625-0) contains supplementary material, which is available to authorized users.

## Background

Virus-induced gene silencing is an antiviral defense mechanism in plants, in which the vital elements involved are virus-derived small interfering RNAs (vsiRNAs) which are mainly 20–24 nucleotides (nt) in length [1, 2]. The vsiRNAs are produced by Dicer-like proteins (DCLs) from viral double-stranded RNA (dsRNA) replication intermediates during the viral replication process, or from highly structured single-stranded RNA molecules present in virus-infected host plants [1–4]. These vsiRNAs are templates generated in host cells that are loaded into Argonaute proteins (AGOs)-containing RNA-induced silencing complexes (RISCs), which then guide the target genomic and subgenomic viral mRNA in a sequence-specific manner to interfere with virus replication, translation and movement, and, in some cases, eliminate the viral infection [3, 5–11].

It is well known that DCLs, AGOs, and the RNA-dependent RNA polymerases (RDRs) participate in the antiviral silencing pathways as key silencing factors, and their RNA silencing activities are varied under different temperatures [4, 12–15]. In the model plant *Arabidopsis*, among the four relatively well-studied DCLs, DCL4 and DCL2 function in RNA silencing against RNA viruses by producing 21- and 22-nt vsiRNAs, respectively. It has been demonstrated that the activity of DCL2 in producing specific 22-nt vsiRNAs derived from *Turnip crinkle virus* (TCV) in *Arabidopsis* is enhanced by higher temperatures [16, 17]. DCLs-generated vsiRNAs are associated with specific AGO complexes, a process partially dependent on the 5'-terminal nucleotides. For instances, vsiRNAs with a 5'-terminal uridine or adenosine are recruited preferentially by AGO1 and AGO2 [18]. Recent studies have also shown that AGO2 plays an antiviral role in the temperature-dependent survival of TCV- and *Potato virus X* (PVX)-infected *Arabidopsis* plants [17, 19, 20]. In addition, RDRs like RDR1, RDR2, or RDR6 are involved in the biogenesis of secondary vsiRNAs to further enhance the antiviral RNA silencing efficiency [21–23]. In *N. benthamiana*, silencing of *RDR6* increases viral RNA accumulation and facilitates viruses to invade the meristem tissue [21]. High temperatures intensify the RDR6 activity in the antiviral RNA-silencing defense response [15]. It has been documented that RDR6 plays a tissue-specific role in the inhibition of *Chinese wheat mosaic virus* (CWMV) accumulation and vsiRNA biogenesis at higher temperatures [7, 15, 24, 25].

*Apple stem grooving virus* (ASGV), a member of the genus *Capillovirus* in the family *Betaflexiviridae* [26]. The ASGV genome is a positive-sense ssRNA with 6.5 kb in length that contains two overlapping open reading frames (ORFs). The larger ORF1 encodes a polyprotein of 240 kDa, in which the N-terminal region contains replicase domains including methyltransferase (Met), papain-like protease (P-pro), NTP-binding helicase (Hel), and the RNA dependent RNA polymerase (RdRp), and the C-terminal region is the coat protein (CP) of 27 kDa [27]. ORF2 is embedded within the ORF1 and encodes a movement protein (MP) of approximately 36 kDa [27]. MP and CP may be produced through the 3′-coterminal subgenomic RNAs (sgRNAs), and CP expression from sgRNA is essential for ASGV systemic infection in the host [28, 29]. Phylogenetic analysis of 16 ASGV full-length genomic sequences clusters them into two groups with no correlations to host and geographical origins [30, 31]. ASGV infection is symptomless on most commercial cultivars of apple and pear, but does induce the typical symptoms of stem pitting and grooving on some cultivars of citrus, lily, kiwifruit, and pear [32–35]. In asymptomatic apple plantlets, ASGV infection induces global gene expression changes, suggesting that extensive host genome-wide gene expression changes do not necessarily lead to disease symptoms [36]. In pear, ASGV infection often deteriorates fruit quality [37]. In the past several years, an increasing incidence of ASGV infection was observed in the pear-growing areas of China, leading to substantial economic losses [37, 38]. High temperature in combination with shoot meristem tip culture is an effective way to obtain virus-free germplasm to control viral diseases of fruit trees [39, 40]. The absence of viruses in the shoot meristem tip tissues is of practical importance because virus-free clones can be generated from infected shoots by culturing excised meristem tips. The effect of temperature on the RNA-silencing activities in plants has been investigated. Accumulated evidence suggests that low temperature inhibits RNA silencing-mediated defense by limiting the generation of small interfering RNA (siRNA) molecules, and high temperature promotes this innate immunity via increasing siRNA accumulation levels [15, 41–43]. In virus-infected plants, viral symptoms disappear in new leaves at high temperatures, resulting from the higher temperature-mediated acceleration of the host antiviral gene silencing system in the meristem tip [9, 39, 44]. In a recent study, we found that thermotherapy elimination of ASGV from Asian pear is associated with the high temperature-induced mixed action of a number of miRNA-mediated target genes related to disease defense and hormone signal transduction pathways in the apical meristem of pear shoots [45]. These data suggest that elevated temperatures may enhance vsiRNA-mediated antiviral gene silencing activity, which in turn reduces the accumulation of viral RNA in the infected meristem tip cells of in vitro-cultured pear shoots.

To explore the possible roles of vsiRNA in the interaction of ASGV and pear plants at higher temperatures, we determined the complete genomic sequence of the ASGV-Js2 isolate and profiled the vsiRNAs in the meristem tip of in vitro-cultured pear shoots at 24 and 37 °C. We analyzed the distribution of vsiRNAs along the viral genome. The corresponding accumulation levels in relation to viral titer and the expression profiles of vsiRNA were also characterized in the shoot tip tissues by RT-qPCR. Furthermore, we determined the *PpDCL2,4*, *PpAGOs*, and *PpRDR1* mRNA sequences and assessed their relative accumulation levels in the ASGV-infected pear shoot tips at 24 and 37 °C. Collectively, these results assist in a better understanding of the roles of vsiRNAs in ASGV infection in vitro-grown pear meristem tips in response to high temperature.

## Methods

### Plant material

In vitro-grown *P. p*yrifolia cv. ‘Jinshui no. 2’ shoots infected with ASGV or ASGV-free, were confirmed by RT-PCR (Additional file 1: Figure S1). ASGV-infected and ASGV-free ‘Jinshui no. 2’ shoots ~1 cm in length were cut and transferred to MS medium, and treated at a 37 °C thermotherapy chamber (16 h light of 1500 lux and 8 h dark) or cultured at 24 °C as controls.

### Bioinformatics analysis of vsiRNA sequences

Clean small RNA reads were obtained from previously constructed sRNA libraries derived from T24 and T37, representing the mixed samples containing equal amounts of total RNAs isolated from the pear shoots treated for 1 and 5 days at 24 and 37 °C, respectively [45]. The small RNA reads were then aligned to the ASGV-Js2 genome (serving as a reference sequence) obtained in this study (see below). The vsiRNA profiles along the viral sense and antisense genome were determined using the Perl scripts and Bowtie software with a stringency allowing no more than two mismatches per read [46, 47].

### Amplification and cloning of the full-length ASGV genome from *P. pyrifolia* cv. ‘Jinshui no. 2’ shoots

The full-length genome sequence of the ASGV-Js2 isolate sequence was determined by standard PCR with specific primers (Additional file 2: Table S1) designed based on the full-length genomic sequences of 17 previously reported ASGV isolates, followed by 5'-RACE and 3'-RACE (Takara Biotechnology Company, Dalian, China) to obtain the 5'- and 3'-terminal regions of the viral genomic RNA. The PCR products were gel-purified and cloned into the pMD18-T vector (Takara). At least three independent overlapping clones in both orientations were sequenced at Jinsirui Biotechnology and Service Co. Ltd (Nanjing, Jiangsu province, China). The full-length sequence of the virus isolate was assembled using clones derived from overlapping RT-PCR fragments by the program Vector NTI 10.0 (Invitrogen, USA).

### Identification and cloning of *PpDCL2,4, PpAGO1,2,4*, and *PpRDR1* sequences from *P. pyrifolia*

Primers used for amplification were designed based on the pear (*Pyrus bretschneideri* Rehd.) genome database [48] using Oligo7 [49] and are listed (Additional file 3: Table S2). *PpDCL2,4*, *PpAGO1,2*,*4*, and *PpRDR1* sequences were amplified by RT-PCR using specific primers from total RNA as template. The PCR products were purified and cloned. The *PpDCL2,4, PpAGO1,2,4,* and *PpRDR1* DNA sequences were deposited into GenBank and their accession numbers are given in Additional file 3: Table S2.

### Phylogenetic tress *S*equence analysis

Sequence similarity searches were performed with the NCBI BLASTN and BLASTX online programs. Pairwise alignments of nucleotide and amino acid sequence were made to determine sequence identity and similarity. Phylogenetic analyses based on multiple sequence alignments of the nucleotide sequences and the predicted protein sequences, respectively, were performed using the Neighbour-joining (NJ) method incorporated in the programs Clustal X 1.83 and MEGA6 software [50, 51].

### Quantitative real-time PCR analyses

To detect the vsiRNA expression levels obtained from the high-throughput sRNA sequencing, real-time quantitative PCR (RT-qPCR) was performed by One Step microRNA Prime-Script sRNA cDNA Synthesis Kit with Poly (A) tails and a special oligo-dT adaptor according to supplier’s instructions (Takara) as described by Liu et al. [45]. Total RNAs from 5 mm ASGV-infected or virus-free meristem tips cultured at 37 and 24 °C for 1 and 5 days were extracted using the CTAB method [45, 52]. All specific vsiRNA forward primers were designed based on the mature vsiRNA sequence (Additional file 4: Table S3). In addition, the expression levels of ASGV *mp*, *PpDCL2,4*, *PpAGO1,2,4*, and *PpRDR1* were analyzed by real-time PCR. Two or three sets of primers were designed and estimated for each gene. The best primer set for each gene is provided in Additional file 4: Table S3. Each sample was a mixture of equal amounts of total RNA from the ASGV-infected or virus-free shoots treated at 24 and 37 °C for 1 and 5 days. RNA samples were digested with DNAase I, reverse-transcribed into cDNA, and then used as templates for RT-qPCR as described previously [45]. The *Actin* gene was used as an internal reference gene for normalization of ASGV *mp*, *PpDCL2,4*, *PpAGOs*, and *PpRDR1* gene expression levels. The RT-qPCR for each gene was performed for three replicates. The experimental data were analyzed essentially as described previously [45, 53].

## Results

### Cloning and sequencing of the ASGV-Js2 genome from in vitro-grown shoots of *P. pyrifolia* cv. ‘Jinshui no. 2’

**Fig. 1:**
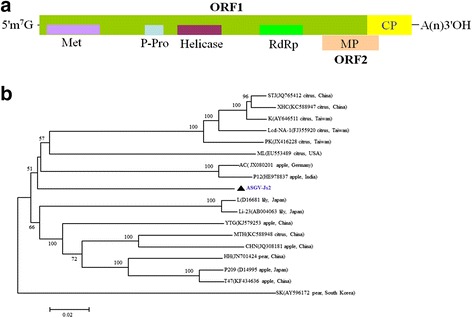
ASGV-Js2 genome analysis. **a** Diagram showing the organization of the ASGV-Js2 genome; **b** Phylogenetic analysis of ASGV full-length genomic sequences from the ASGV-Js2 isolate used in this study with 17 previously-reported isolates. Isolate names are followed by the GenBank accession number, the plant host, and the country of origin in parentheses. The tree was reconstructed by the Neighbour-joining method (NJ) with 1,000 bootstrap replicates, and bootstrap values >50 % are shown at branch nodes. Bars represent 0.02 substitutions per site

To profile vsiRNAs derived from the ASGV-Js2 isolate in pear, we needed to determine the full-length genome sequence of the ASGV-Js2 isolate. The full-length ASGV-Js2 genome was cloned and sequenced as described in Methods (Additional file 2: Table S1). The genomic sequence was deposited into GenBank (accession no. KU198289). The ASGV-Js2 viral genome is 6,497 bp in length and contains two overlapping ORFs (ORF1 and ORF2) (Fig. 1a), similar to the data reported previously [27, 28]. A phylogenetic analysis of ASGV-Js2 and 17 additional full-length ASGV genomic sequences available in GenBank showed that ASGV-Js2 defines a unique cluster, and the pairwise nucleotide identities range from 80.0 to 83 % within this phylogenetic ASGV group (Fig. 1b and Additional file 5: Table S4).

### Characteristics of the ASGV-derived vsiRNA population in *P. pyrifolia* shoot tips in response to high temperature

**Fig. 2:**
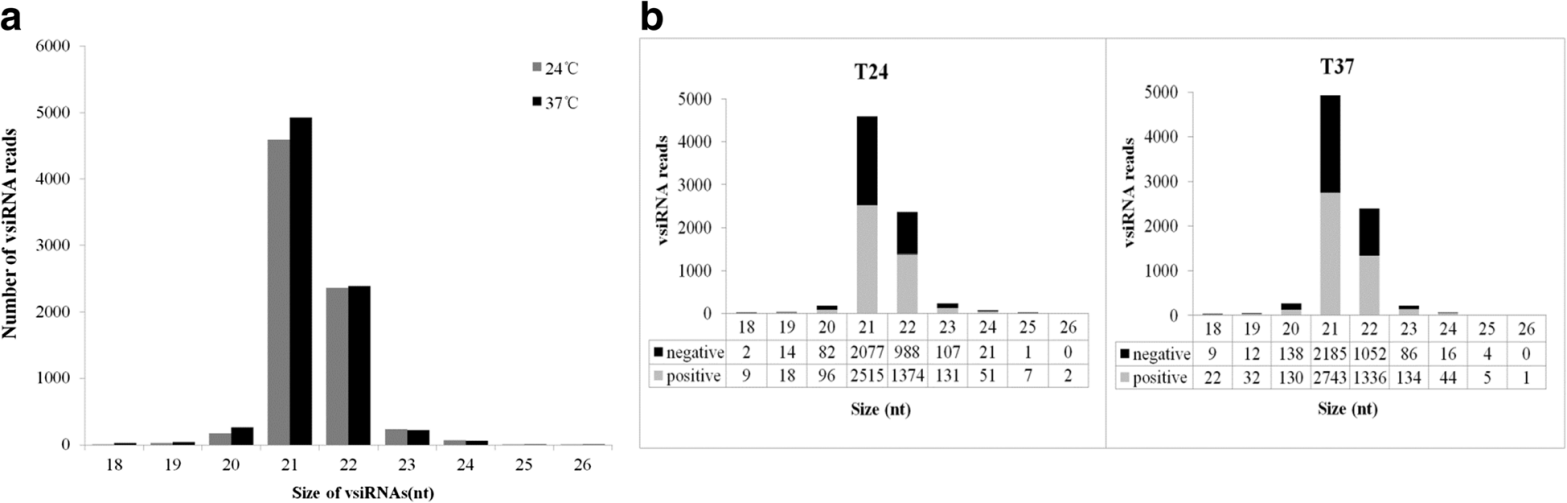
Analysis of the ASGV-derived vsiRNA population in *P. pyrifolia* shoots. **a** Distribution of ASGV-Js2-derived vsiRNA lengths from *P. pyrifolia* shoots cultured in vitro at 24 °C and 37 °C. The numbers of siRNAs (18–26 nt) in the 24 and 37 °C libraries are shown in gray and blank, respectively. **b** Distribution of the numbers of sense and antisense vsiRNAs reads in the 24 and 37 °C libraries. The positive and negative vsiRNAs reads mapped to strands of the virus are represented in gray and blank, respectively. Histograms represent the numbers of sense and antisense vsiRNA reads in each size class

Small RNAs obtained from previously constructed libraries of in vitro-cultured *P. pyrifolia* shoots infected by ASGV-Js2 at 24 and 37 °C were subjected to deep-sequencing on an Illumina platform by Liu et al. [45]. To determine whether siRNAs identified in pear derived from ASGV-Js2 are responsive to high temperature treatment, the generated siRNA sequences were aligned against the ASGV genomic and the negative-sense genomic RNA sequences. A total of 7,495 and 7,949 reads ranging from 18 to 26 nt in length were identified to be ASGV-derived siRNAs from the 24 and 37 °C libraries, respectively. These vsiRNAs were mapped to the ASGV-Js2 genome sequence. An analysis of the length distribution of the vsiRNAs showed that the 21-nt vsiRNAs were the most abundant, representing 61 % (4,592 reads) and 62 % (4,928 reads) of total vsiRNAs from the 24 °C- and 37 °C-treated ASGV-infected pear shoot libraries, respectively (Fig. 2a). The next most abundant class was the 22 nt molecules, accounting for 31.5 % (2,362/7,495 reads) and 30 % (2,388/7,949 reads) of total vsiRNAs from the 24 and 37 °C libraries, respectively (Fig. 2a). Relatively more vsiRNAs (4,203 from the 24 °C library and 4,447 from the 37 °C libraries) were found to be derived from the positive strand (Fig. 2b). Fewer vsiRNAs (3,292 and 3,502) from both libraries were derived from the negative strand (Fig. 2b). The ratio of sense/antisense 21-nt v siRNAs was 1.21 and 1.25 for the 24 and 37 °C libraries, respectively (Fig. 2b). The relatively high proportion of 21- and 22-nt vsiRNAs confirmed the presence of DCL4- and DCL2-like Dicer ribonucleases in pear shoot tips that target viral RNAs to mediate vsiRNA biogenesis.

**Fig. 3:**
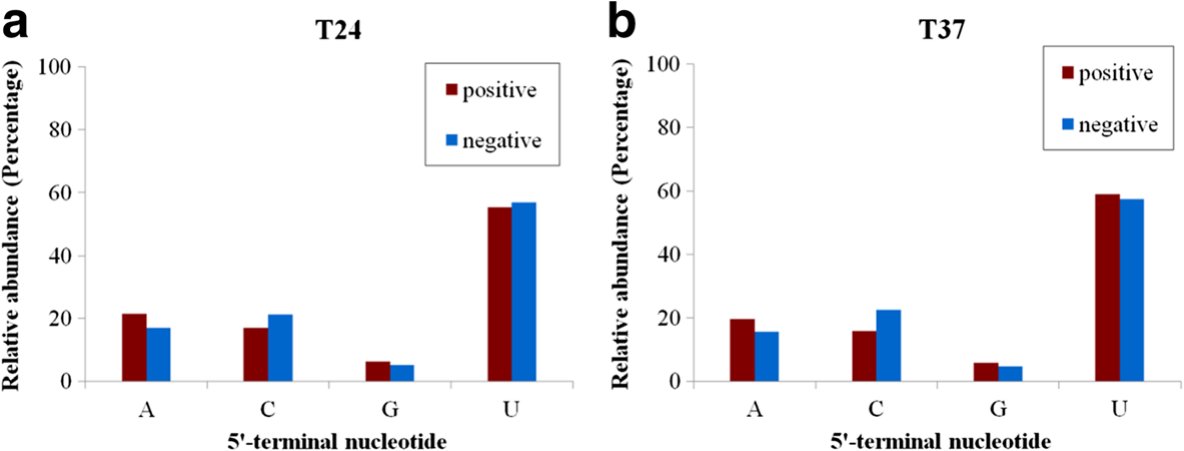
Relative frequencies of the 5'-terminal nucleotide in pear vsiRNAs. The positive- and negative-strand vsiRNAs were derived from ASGV-Js2-infected in vitro-grown *P. pyrifolia* shoots at 24 °C (**a**) and 37 °C (**b**)

**Fig. 4:**
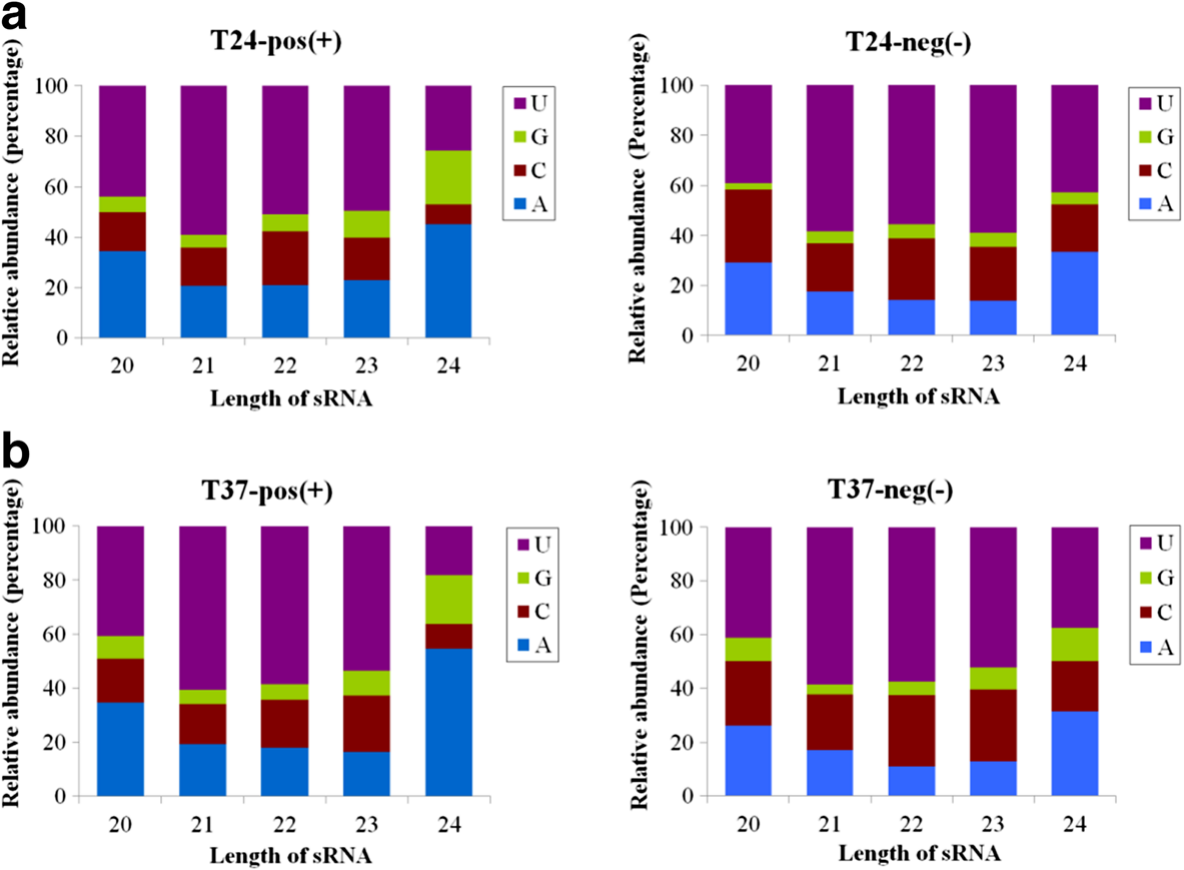
Characterization of the vsiRNA 5'-terminal nucleotides. The relative abundance of the four 5'-terminal nucleotides are shown for the 20 to 24 nt vsiRNAs. The genomic and anti-genomic ASGV-derived vsiRNAs were from in vitro-grown *P. pyrifolia* shoot tips at 24 °C (**a**) and 37 °C (**b**)

Previous data have shown that the 5'-terminal nucleotides partially influence the loading of siRNAs into specific AGO complexes in *Arabidopsis*, and may have roles in targeting vsiRNAs to AGOs [18]. In this study, we analyzed the relative frequencies of the 5'-terminal nucleotides of vsiRNAs from the 24 and 37 °C libraries in an effort to understand potential interactions between vsiRNAs and AGOs in pear. Among all the vsiRNAs, U was found to be the most common nucleotide at the 5' end, with 55.32 % and 56.92 % derived from the ASGV positive and negative strands from in vitro-grown pear shoots at 24 °C, respectively (Fig. 3a). U was also the most abundant 5' terminal nucleotide (58.88 and 57.35 % corresponding to the ASGV positive and negative strands, respectively) in the 37 °C-treated shoots (Fig. 3b). In addition, detailed analyses of the relative frequencies of the 5'-terminal nucleotides for the 20 to 24-nt vsiRNAs revealed that the vsiRNAs ranging from 20 to 23-nt showed a preference for U, with 43.75 to 59.09 % and 40.77 to 60.77 % of those derived from the genomic ASGV RNA in the 24 and 37 °C libraries, respectively. However, the 24-nt vsiRNAs showed a preference for A at the 5' end, with 45.10 and 54.55 % in the 24 and 37 °C libraries (Fig. 4). Also, the 20 to 24-nt vsiRNA had a preference for U with 39.02–58.89 and 37.50–58.63 % derived from the negative ASGV strand in the 24 and 37 °C libraries, respectively (Fig. 4). These results support the idea that the 21- and 22-nt vsiRNAs with U as the 5'-terminal nucleotide might be preferentially loaded into AGO1 [18], consistent with the role described for AGO1 in defending against RNA virus infection in *Arabidopsis* [24].

### Distribution of ASGV-derived siRNA abundance in *P. pyrifolia* shoot tips in response to high temperature

**Fig. 5:**
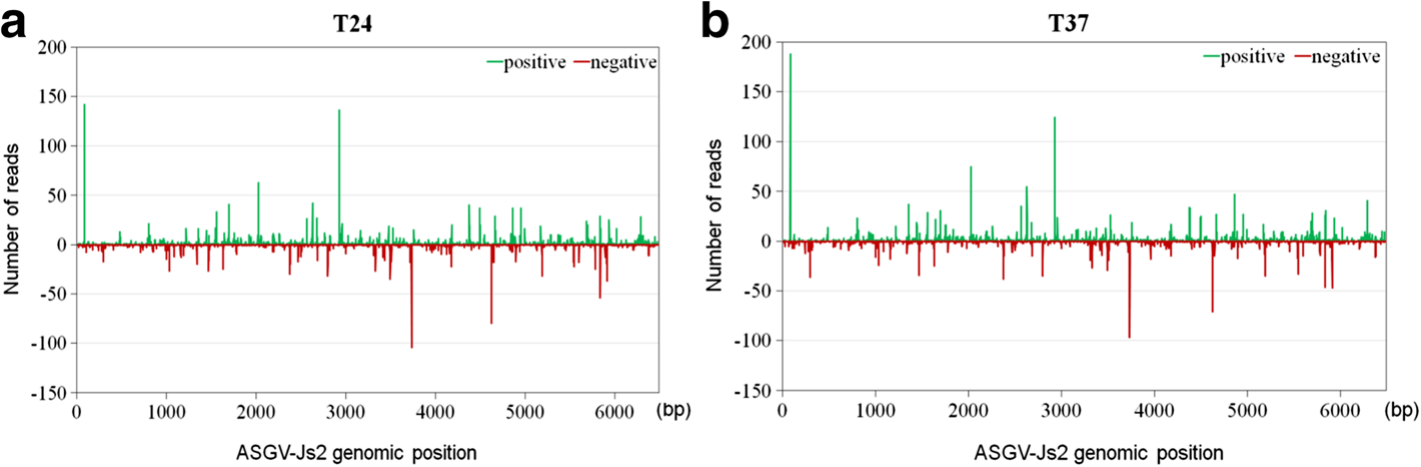
Profiles of variant-specific sRNA reads along the ASGV-Js2 genome. The genomic positions of vsiRNA reads mapped to the ASGV-Js2 genome are shown. **a** and **b** show the profiles of the ASGV-derived vsiRNAs mapped to the ASGV-Js2 genome from the 24 and 37 °C libraries, respectively. Reads that mapped to the positive or negative strands of the ASGV-Js2 genome are represented in green and red, respectively

To determine the distribution pattern of the vsiRNAs along the ASGV-Js2 genome, vsiRNA sequences were mapped to the viral genome. The results showed that the vsiRNAs were distributed unevenly along both strands of the ASGV-Js2 genome, implying there are variations in the relative abundance of siRNAs targeting different regions of the ASGV-Js2 genome in the 24- and 37 °C-treated pear shoots (Fig. 5a and b). No apparent vsiRNA-production hotspots in the ASGV-Js2 genome were found from our analyses.

**Fig. 6:**
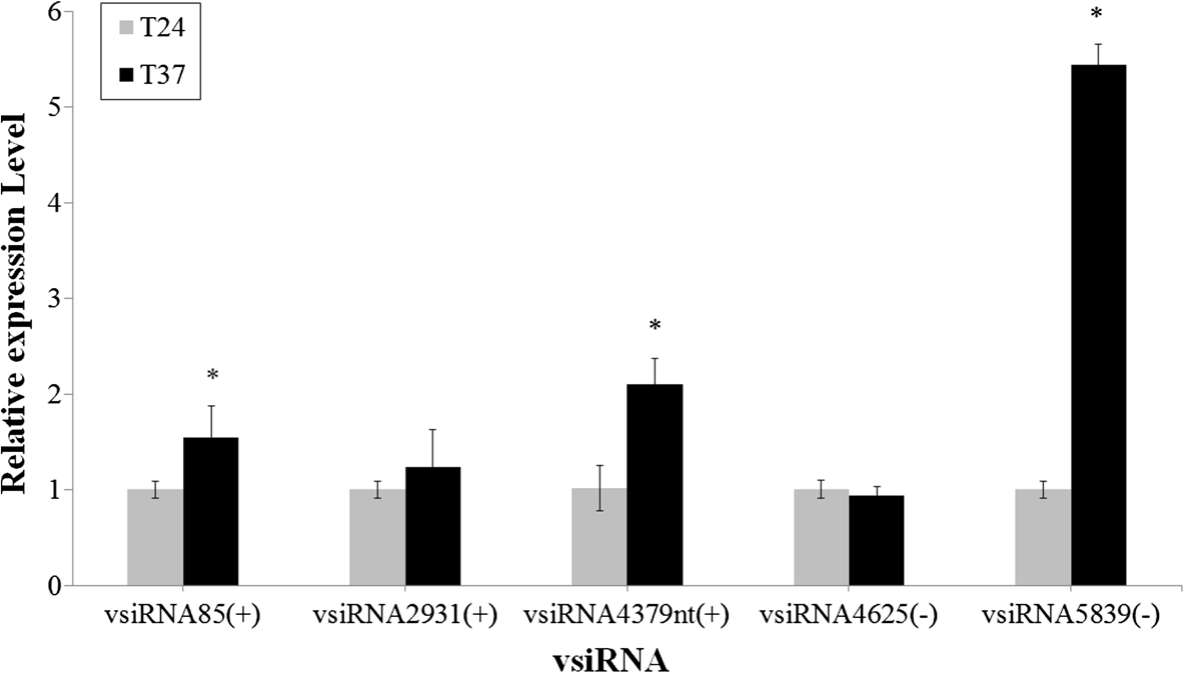
Profiles of vsiRNAs derived from the positive and negative strands of the ASGV genome by qRT-PCR. Error bars indicate standard deviations. Asterisks represent significant differences

To validate the existence of the predicted vsiRNAs in the pear shoot meristem tip using small RNA sequence analysis, some vsiRNAs derived from different ASGV-Js2 genomic regions in the two libraries were analyzed by RT-qPCR. These vsiRNAs matched the ASGV-Js2 genome positive strand at positions 85–105, 2931–2951 and 4379–4399, designated vsiRNA85(+), vsiRNA2931(+), and vsiRNA4379(+), respectively. Similarly, some reads were mapped to the negative strand at positions 5839–5859 and 4625–4645, designated vsiRNA5839(−) and vsiRNA4625(−), respectively. RT-qPCR results discovered that the expression levels of vsiRNA2931(+) and vsiRNA4625(−) in the shoot meristem tip tissue at 37 °C were nearly equal to those at 24 °C (Fig. 6). In contrast, the expression levels of vsiRNA85(+), vsiRNA4379(+) and vsiRNA5839(−) were elevated at 37 °C, when compared to those from the 24 °C-treated shoot tips (Fig. 6).

### The effects of high temperature on ASGV infection in *P. pyrifolia* shoot tips

**Fig. 7:**
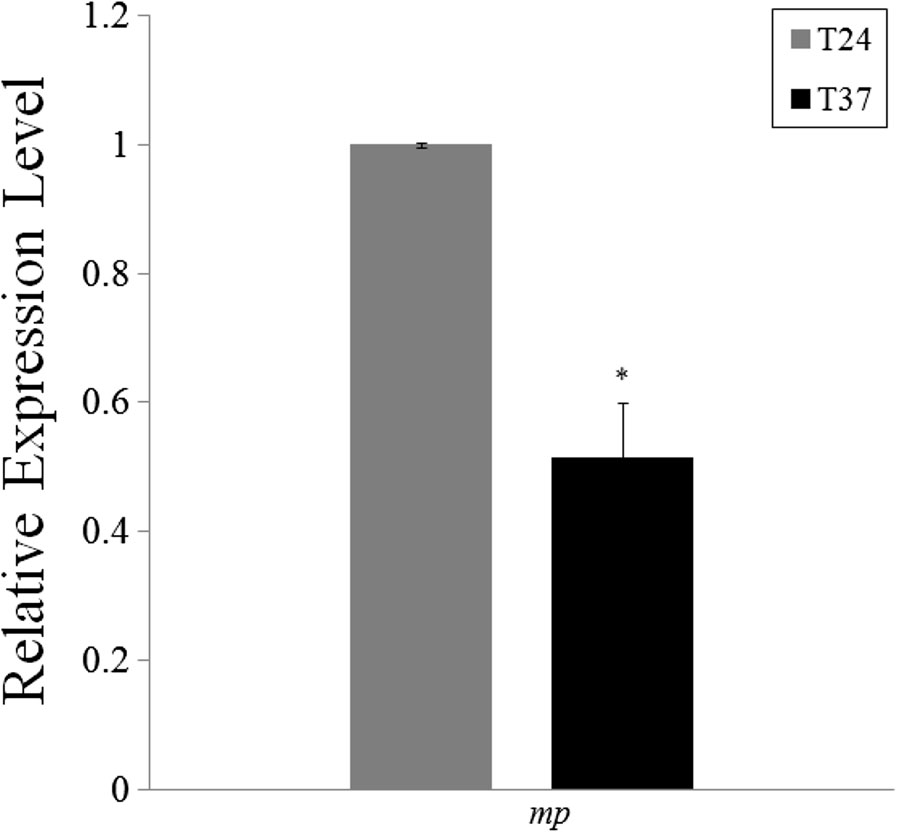
Relative accumulation of ASGV-Js2 genomic RNA and MP subgenomic RNA in tip tissues of in vitro-grown pear shoots in response to 37 °C treatment. Error bars indicate standard deviations. Asterisks represent significant differences

ASGV infection may be evaluated by quantification of the level of ASGV genomic RNA and subgenomic RNA. As mentioned earlier, MP may be produced via subgenomic RNA. To investigate the effect of high temperature on ASGV infection in the meristem tip of in vitro-grown *P. pyrifolia* shoots, we determined the ASGV genomic RNA and its MP subgenomic RNA accumulation at 24 and 37 °C by RT-qPCR. The level of viral genomic RNA and MP subgenomic RNA was found to decrease by 50 % in response to the 37 °C treatment compared to 24 °C (Fig. 7). This indicated that high temperature drastically inhibited ASGV infection in the pear meristem tip.

### Expression profiles of *PpDCL2, PpDCL4*, *PpAGO1, PpAGO2, PpAGO4*, and *PpRDR1* mRNAs in ASGV-infected *P. pyrifolia* shoot tips at high temperature

**Fig. 8:**
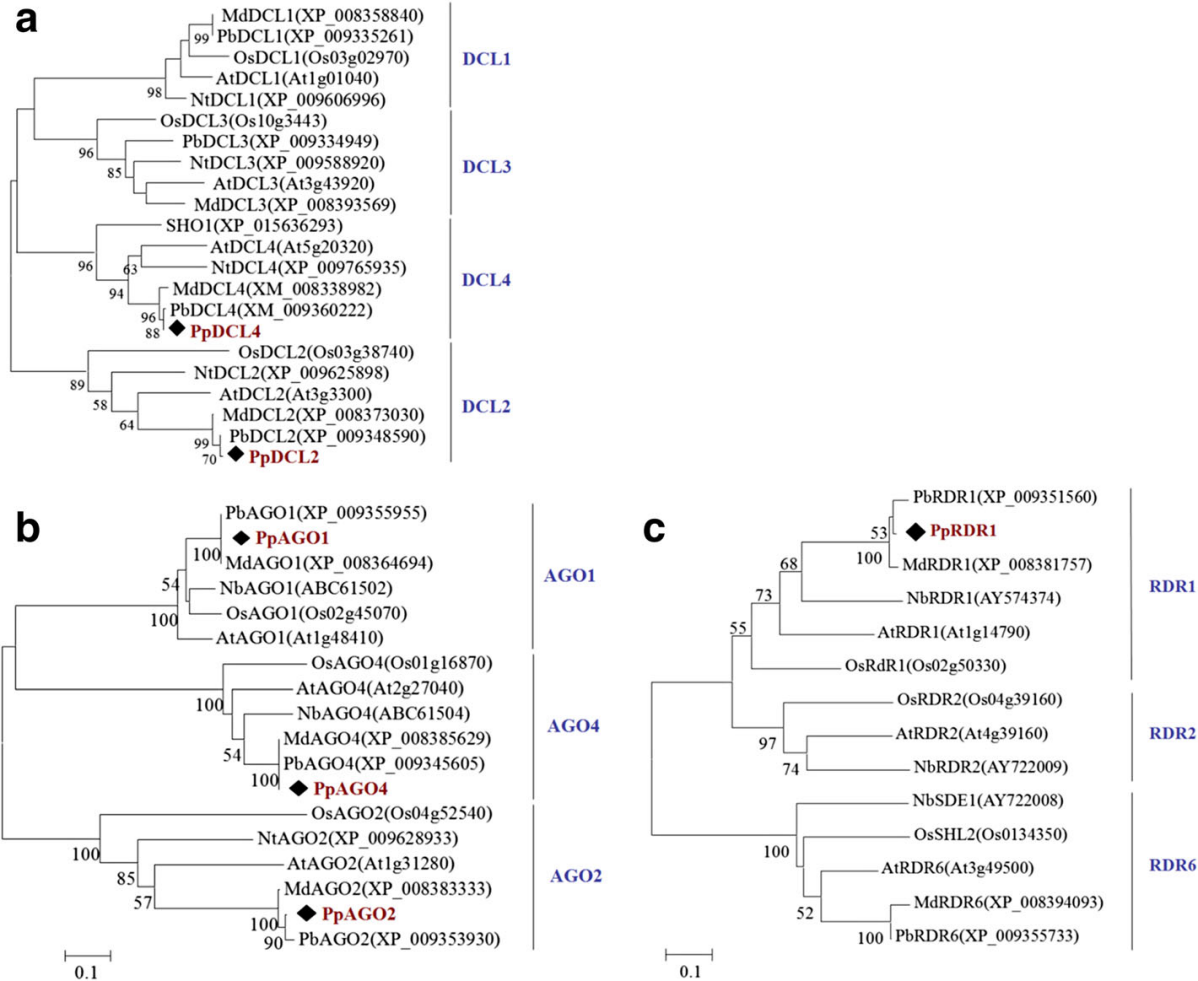
Phylogenetic relationships between the PpDCL2,4, PpAGO1,2,4, and PpRDR1 proteins of *P. pyrifolia* with their homologues in *Pyrus bretschneideri*, *Malus*, *Oryza sativa*, *Nicotiana*, and *Arabidopsis* species. **a**, **b**, and **c** are unrooted Neighbor-joining trees constructed with predicted protein sequences for Dicer-like, Argonaute, and RNA-dependent RNA polymerases from this study and homologous sequences from other isolates deposited into GenBank (gene name followed by GenBank accession number). Statistical analysis was performed with 1,000 bootstrap replicates, and bootstrap values >50 % are shown at branch nodes. Bars represent 0.1 substitutions per site. The predicted *P. pyrifolia* DCL, AGO, and RDR proteins are shown in red in each group. The abbreviations for the AGO, DCL, and RDR proteins used in the phylogenetic trees are as the follows: Pb, *Pyrus bretschneideri*; Pp, *Pyrus pyrifolia*; Md, *Malus domestic*; Os, *Oryza sativa*; Nb, *Nicotiana benthamiana*; Nt, *Nicotiana thaliana*; At, *Arabidopsis thaliana*

In plants, the generation of small RNAs mainly depends on the activities of DCL, AGO, and RDR. Dicer and AGOs are the key factors involved in recognition of dsRNA and degrading target viral RNAs, whereas RDRs mediate the secondary accumulation of siRNAs through *de novo* synthesis [2, 4]. To study the expression of these genes in the ASGV-infected pear shoot tips in response to high temperature treatment, we cloned partial cDNA sequences of *PpDCL2*, *PpDCL4*, *PpAGO1, PpAGO2, PpAGO4*, and *PpRDR1* mRNA and the resulting sequences were deposited into the GenBank database under accession numbers provided in Additional file 3: Table S2. Phylogenetic tree analysis showed that the deduced amino acid sequences of the partial *PpDCL2*, *PpDCL4*, *PpAGO1, PpAGO2, PpAGO4*, and *PpRDR1* cDNA sequences share high levels of sequence conservation with their counterparts from *Pyrus bretschneideri*, *Malus*, *Arabidopsis*, *Nicotiana*, and *Oryza sativa* (Fig. 8a, b and c). These data support the notion that *PpDCL2*, *PpDCL4*, *PpAGO1, PpAGO2, PpAGO4*, and *PpRDR1* genes in *P. pyrifolia* are homologs of the corresponding genes from *Nicotiana, Arabidopsis*, and *Oryza sativa,* and thus may function like DCLs, AGOs, and RDRs as demonstrated in those model plant species [2, 16, 17, 20].

**Fig. 9:**
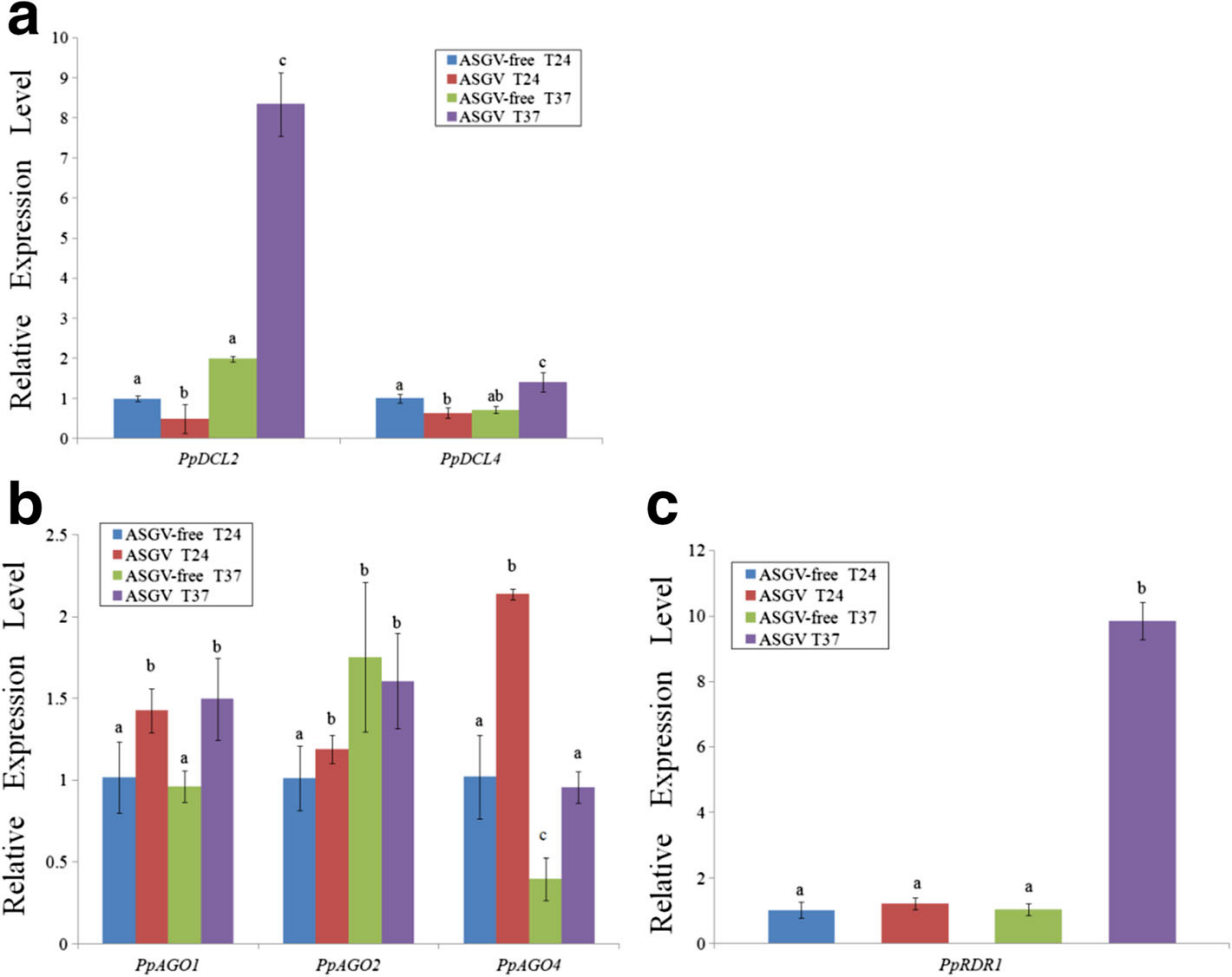
Relative expression levels of *PpDCL2* and *PpDCL24* (**a**), *PpAGO1, PpAGO2,* and *PpAGO4* (**b**) and *PpRDR1* (**c**) genes were determined from total RNA isolated from ASGV-infected and uninfected *P. pyrifolia* shoot tips at 24 and 37 °C by RT-qPCR. Error bars indicate standard deviations. Bars in each histogram labeled with the same letters are not significantly different

Based on the cloned cDNA sequences of *PpDCL2*, *PpDCL4*, *PpAGO1, PpAGO2, PpAGO4*, and *PpRDR1*, primers were designed for RT-qPCR to determine their expression levels (Additional file 4: Table S3). We found that *PpDCL2 and PpDCL4* mRNA increased dramatically in the ASGV-infected pear shoots at the higher temperature, as compared to the controls (Fig. 9a). Similarly, *PpRDR1* expression was also up-regulated in ASGV-infected pear shots at 37 °C (Fig. 9c). Interestingly, it is ASGV infection rather than high temperature treatment significantly up-regulated *PpAGO1* and *PpAGO4* expression (Fig. 9b). In contrast, high temperature did induce higher levels of *PpAGO2*-specific mRNA accumulation (Fig. 9b).

## Discussion

Viral infection triggers the host gene silencing response, leading to vsiRNA accumulation [54–60]. Temperature plays a key role in the RNA silencing-mediated antiviral defense in plants due to its effect on the control of siRNA generation [15, 17, 42, 43]. Although vsiRNAs associated with virulent virus infections under various environmental conditions have been relatively well studied in model plant species, their involvement in latent virus infection in woody plants, especially fruit trees remains poorly characterized [61, 62]. Our study is the first report on the characterization of a population of vsiRNAs in pear plants infected by a latent virus, ASGV-Js2, in response to high temperature treatment.

In this study, small RNA sequencing data revealed the presence of vsiRNAs in the tip of the ASGV-infected *P. pyrifolia* shoots (Figs. 2, 3 and 4). We found that approximately 0.05 % (7,495/13,741,468 reads) and 0.06 % (7,949/14,071,933 reads) reads from the ASGV-infected samples matched the ASGV-Js2 genome from the 24 and 37 °C libraries, respectively. The results revealed that vsiRNAs accounted for a relatively small proportion of small RNAs in the ASGV-infected pear shoots when compared to the levels of vsiRNAs in other virus-host pathosystems in which virus-infected leaves are often used as materials for siRNA profiling studies [11, 57]. Therefore, the low levels of ASGV-derived vsiRNAs may be attributed to the meristem tips of in vitro-cultured pear shoots used in this study. It is well known that there are endogenous restrictions preventing viral genomes from moving into plant meristems. In this study, we also found a continuous and uneven distribution of plus- and minus-sense vsiRNAs throughout the ASGV-Js2 genome in pear at either 24 or 37 °C (Figs. 2b and 5). This is different from findings from several previous reports that vsiRNAs are increasingly distributed toward the 3' end of the viral genome [61–63]. It is not clear if the distribution pattern of vsiRNAs found in this study is also related to the particular tissues, e.g., pear shoots used in this study.

Real-time PCR analysis using primers specific for the MP coding region (Fig. 7) or RdRp sequence (data not shown) demonstrated a reduction in ASGV accumulation in the pear meristem tissues in response to high temperature treatment, similar to what was found in our previous study [45]. Also the accumulation of vsiRNA4379 (+) and vsiRNA5839 (−) derived from the RdRp and CP regions of the ASGV genome increased accordingly (Figs. 5 and 6). These data suggest that vsiRNA abundance is negatively correlated with the levels of the ASGV viral RNA in response to high temperature (37 °C) treatment. This is consistent with the results obtained using other virus-host pathosystems [15]. An exemption is the case of a DNA virus, *Cucurbit leaf crumble virus* (CLCV). The relative abundance of CLCV-derived siRNA is apparently positively correlated with viral titers in pumpkin [55]. A possible explanation for this discrepancy is that each virus-host combination might reflect unique characteristics; specifically, a dynamic equilibrium established during viral infection may affect sRNA levels in different virus-host systems [54, 55, 57, 64]. It has been demonstrated that DCLs, RDRs, AGOs and other factors involved in RNA silencing also participate in antiviral defense in model plant species such as *Arabidopsis,* tobacco, and rice [2, 13, 15, 20, 23, 25, 65–67]. To explore the association of the corresponding homolog proteins with ASGV infection in pear in response to high temperature treatment, we cloned and obtained the partial sequences of *PpDCL2, PpDCL4*, *PpAGO1, PpAGO2, PpAGO4*, and *PpRDR1* and determined their relative expression levels (Figs. 8 and 9). Overall these genes were up-regulated in the ASGV-infected pear shoots at 37 °C, which was accompanied with the reduced level of viral RNA (Fig. 7) and the elevated levels of vsiRNAs (Fig. 6). These data support that high temperature treatment may enhance the RNA silencing capacity in the pear meristem tissue via up-regulating the expression of key components of the antiviral pathway to cope with ASGV infection. Future study is directed to elucidate how temperature regulates gene silencing in the ASGV-infected pear shoots and if this is tissue- or ASGV-specific. Such work would help better understand and improve thermotherapy for the effective control of virus diseases in fruit trees.

## Conclusions

This study represents the first report on the characterization of vsiRNA in the in vitro-grown ASGV-infected pear shoots in response to high temperature treatment. The profiles of vsiRNAs showed an uneven distribution along the ASGV-Js2 genome, and that 21- and 22-nt vsiRNAs preferentially accumulated when cultured at higher temperature. ASGV-specific siRNAs from all libraries had a similar distribution of 5'-terminal nucleotides. U was the most frequent among the 5’ terminal nucleotides, and its frequency was slightly higher at 37 °C. The expression levels of the viral *mp* gene and vsiRNAs were characterized by RT-qPCR. We also cloned *PpDCL2,4*, *PpAGO1,2,4* and *PpRDR1* partial sequences and examined their expression patterns, and found their expression levels were up-regulated in the ASGV-infected pear shoots at 37 °C. This up-regulation was accompanied with the reduced level of viral RNA and the elevated levels of vsiRNAs. Taken together these data suggest that high temperature may induce and enhance the RNA silencing capacity in the pear meristem tissue.

## Additional files

Additional file 1: Figure S1. 1.2% agarose gel electrophoresis of RT-PCR products of ASGV-Js2 isolate. M: Marker II (TIANGEN Biotech, Beijing Co., Ltd.), CK+: P. pyrifolia cv. ‘HuangHua’ shoots as positive control, CK-: P .pyrifolia cv. ‘YuanHuang’ shoots as negative control. Lanes 1-6 show amplification products from virus-free plants (A) and ASGV-infected plants (B). (DOC 532 kb)
Additional file 2: Table S1. Oligonucleotide primers used for amplification of the ASGV-Js2 genome (DOC 45 kb)
Additional file 3: Table S2. Oligonucleotide primer sequences and amplicon characteristics for the *PpDCL2,4*, *PpAGO1,2,4,* and *PpRDR1* genes. (DOC 35 kb)
Additional file 4: Table S3. Oligonucleotide primers used for qRT-PCR expression analysis of vsiRNAs, *P. pyrifolia* mRNAs, and the ASGV-Js2 *mp* gene from in vitro-grown shoots of *P. pyrifolia*. (DOC 42 kb)
Additional file 5: Table S4. Pairwise sequence similarities comparing the ASGV-Js2 full-length genome with 17 ASGV isolates sequences from GenBank. (DOC 66 kb)
