# Supplementary material for: Characterization of virus-derived small interfering RNAs in Apple stem grooving virus-infected in vitro-cultured Pyrus pyrifolia shoot tips in response to high temperature treatment
Source: Virol J. 2016 Oct 6;13:166. doi: 10.1186/s12985-016-0625-0 (PMC5053029; doi:10.1186/s12985-016-0625-0)

**Additional file 1: Figure S1.** 1.2% agarose gel electrophoresis of RT-PCR products of ASGV-Js2 isolate. M: Marker II (TIANGEN Biotech, Beijing Co., Ltd.), CK+: *P. pyrifolia* cv. ‘HuangHua’ shoots as positive control, CK-: *P .pyrifolia* cv. ‘YuanHuang’ shoots as negative control. Lanes 1-6 show amplification products from virus-free plants (**A**) and ASGV-infected plants (**B**).


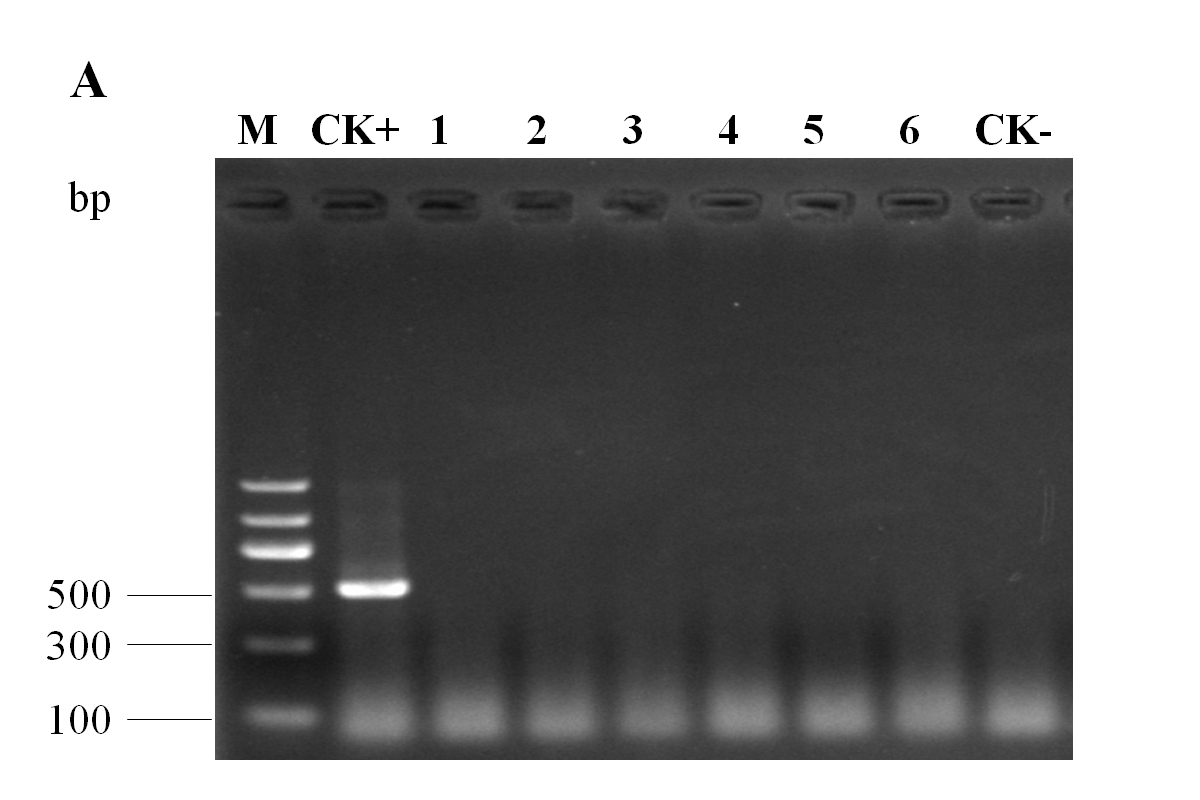

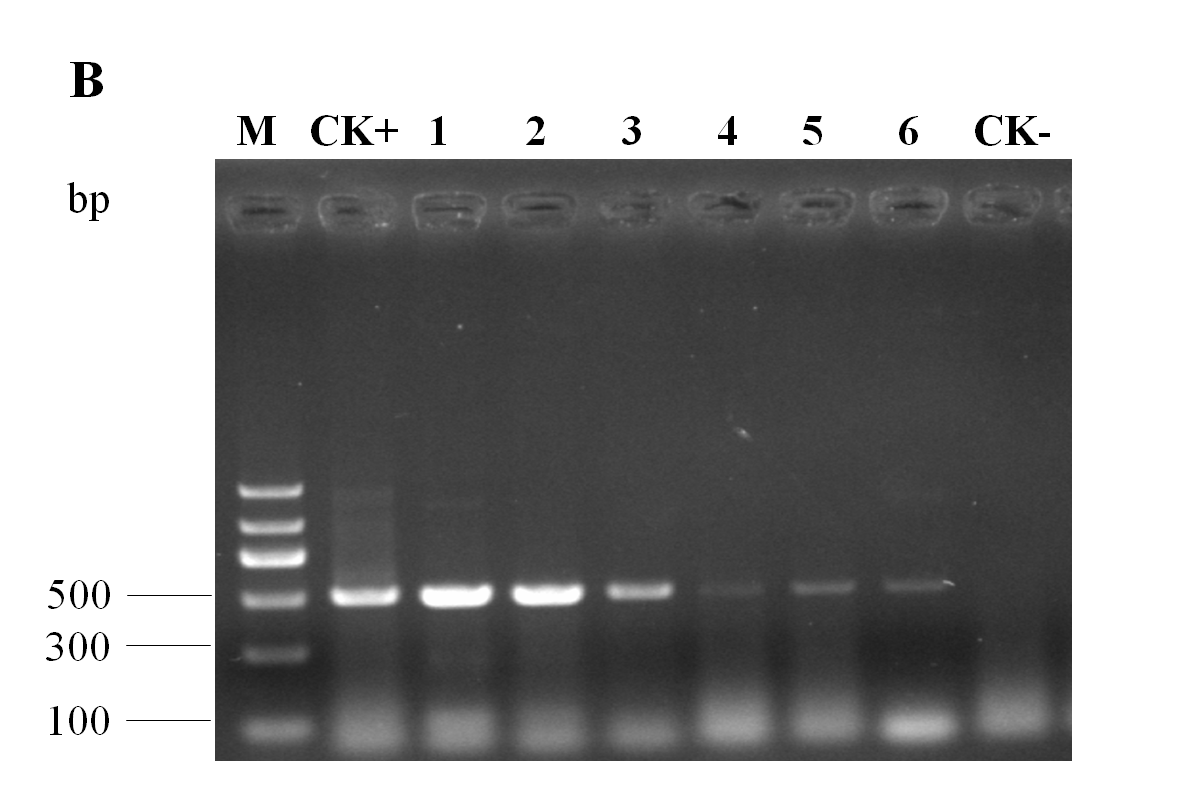

Supplement: Additional file 1: Figure S1. — 1.2% agarose gel electrophoresis of RT-PCR products of ASGV-Js2 isolate. M: Marker II (TIANGEN Biotech, Beijing Co., Ltd.), CK+: P. pyrifolia cv. ‘HuangHua’ shoots as positive control, CK-: P .pyrifolia cv. ‘YuanHuang’ shoots as negative control. Lanes 1-6 show amplification products from virus-free plants (A) and ASGV-infected plants (B). (DOC 532 kb) [file 12985_2016_625_MOESM1_ESM.doc]
