# Supplementary material for: Characterization of virus-derived small interfering RNAs in Apple stem grooving virus-infected in vitro-cultured Pyrus pyrifolia shoot tips in response to high temperature treatment
Source: Virol J. 2016 Oct 6;13:166. doi: 10.1186/s12985-016-0625-0 (PMC5053029; doi:10.1186/s12985-016-0625-0)
Supplement: Additional file 2: Table S1. — Oligonucleotide primers used for amplification of the ASGV-Js2 genome (DOC 45 kb) [file 12985_2016_625_MOESM2_ESM.doc]

**Additional file 2:Table S1.** Oligonucleotide primers used for amplification of the ASGV-Js2 genome

| Primer | Primer Sequence (5'-3') | Positions | Size |
| --- | --- | --- | --- |
| F5-F | GGCTTAATTTCCGCGCTTTA | 13-32 | 1010 |
| F5-R | GACAAGCCATGTATGTACTGAGG | 1000-1022 |
| FD-F | GATAAGAGTTACCAAGTTGCAGA | 823-845 | 358 |
| FD-R | GACCCATTGCAGAATTGATACA | 1159-1180 |
| FA-F | CCTCCCTCAGTACATACATGGC | 996-1017 | 1098 |
| FA-R | TGCAGTACATCTTGCCATTTACTCC | 2069-2093 |
| FC-F | GATGAGCTGCTTGAATGTATTGA | 1934-1956 | 538 |
| FC-R | GCATCCTCCTTGGACTAATGA | 2451-2471 |
| FXB-F | AGCGCAAATGTCAAATGAGAAT | 2164-2185 | 2118 |
| FXB-R | CTCATTATTGCCAGTGAACCGAGT | 4258-4281 |
| F1-F | AAGGAAAGATGTTCACTGAGGC | 3909-3930 | 1014 |
| F1-R | CTTTTGATGCAATTGAGAACGT | 4901-4922 |
| Mp-F | CGATCGTCAATGTCAACCAT | 4793-4812 | 954 |
| Mp-R | GGGAGGAACCGTCAGAAGTT | 5727-5746 |
| Cp3′-F | CACCGGGTAGGAGTATATCTATGG | 5678-5701 | 820 |
| Cp3′-R | AGAGTGGACAAACTCTAGACTC | 6476-6497 |
| 3′RACE-R-outer | TACCGTCGTTCCACTAGTGATTT |  | 3′ RACE Kit provided |
| 3′RACE-R-inner | CGCGGATCCTCCACTAGTGATTTCACTATAGG |  |
| 3′RACE-F-outer | CTTCTAGGCAGAACTCTTTGAACGA | 6044-6068 | 183 |
| 3′RACE-F-inner | CGGAGAGTAACCTGGAACTGGA | 6327-6348 |
| 5′RACE-F-outer | CATGGCTACATGCTGACAGCCTA |  | 5′ RACE Kit provided |
| 5′RACE-F-inner | CGCGGATCCACAGCCTACTGATGATCAGTCGATG |  |
| 5′RACE-R-outer | GGCTAAGGAAATGTAGTTCATCGTG | 451-475 | 318 |
| 5′RACE-R-inner | AAGACAGCAGACTAGGAATGCAG | 292-314 |

Note: Primer position refers to the full-length genomic sequence of the ASGV-J2 isolate.
