# Supplementary material for: Characterization of virus-derived small interfering RNAs in Apple stem grooving virus-infected in vitro-cultured Pyrus pyrifolia shoot tips in response to high temperature treatment
Source: Virol J. 2016 Oct 6;13:166. doi: 10.1186/s12985-016-0625-0 (PMC5053029; doi:10.1186/s12985-016-0625-0)
Supplement: Additional file 3: Table S2. — Oligonucleotide primer sequences and amplicon characteristics for the PpDCL2,4, PpAGO1,2,4, and PpRDR1 genes. (DOC 35 kb) [file 12985_2016_625_MOESM3_ESM.doc]

**Additional file 3:Table S2.** Oligonucleotide primer sequences and amplicon characteristics for the *PpDCL2,4*, *PpAGO1,2,4,* and *PpRDR1* genes.

| Gene name | Primer sequence (5'-3') | Fragment size (bp) | Accession number |
| --- | --- | --- | --- |
| *PpDCL2* | Forward- TTCGTTCTCACTTGTTCCGT  Reverse-CTGGTAACCTCCTGGTATGTAAGA | 727 | KU215387 |
| *PpDCL4* | Forward- AGACGTGCTGCCTTGTGATA | 1037 | KX014745 |
|  | Reverse-GATAGTCCCCCAATCAATGC |  |  |
| *PpAGO1* | Forward-ACTATCCTGTTGCACGTTC  Reverse-AAGGGCAGTTCTATCAAGT | 1568 | KU215388 |
| *PpAGO2* | Forward- GGCACAAAAGCTATCCGAAC  Reverse-GACTGTTGACGGAGAGAAAT | 1231 | KU215385 |
| *PpAGO4* | Forward- ATGAGAGTGACCGTAAGAGAT  Reverse-TTCAGCCTCAGTCCCCAG | 1860 | KU215389 |
| *PpRDR1* | Forward-CTTTGTGGATGAGGACTTGA  Reverse-AAGTTACAATGGAGGAGGTT | 1348 | KU215386 |
