# Supplementary material for: Characterization of virus-derived small interfering RNAs in Apple stem grooving virus-infected in vitro-cultured Pyrus pyrifolia shoot tips in response to high temperature treatment
Source: Virol J. 2016 Oct 6;13:166. doi: 10.1186/s12985-016-0625-0 (PMC5053029; doi:10.1186/s12985-016-0625-0)
Supplement: Additional file 4: Table S3. — Oligonucleotide primers used for qRT-PCR expression analysis of vsiRNAs, P. pyrifolia mRNAs, and the ASGV-Js2 mp gene from in vitro-grown shoots of P. pyrifolia. (DOC 42 kb) [file 12985_2016_625_MOESM4_ESM.doc]

**Additional file 4: Table S3.** Oligonucleotide primers used for qRT-PCR expression analysis of vsiRNAs, *P. pyrifolia* mRNAs, and the ASGV-Js2 *mp* gene from *in vitro*-grown shoots of *P. pyrifolia*.

| Name | | Primers (5'-3') | | Size (bp) | |
| --- | --- | --- | --- | --- | --- |
| vsiRNA Primer sequence | | | | | |
| *vsiRNA 85*(+) | TCCGAGCAAGCAGTCTGAT-Forward | | 19 | | |
| *vsiRNA2931*(+) | GGTCAGAACTGAGGATTACGGA-Forward | | 22 | | |
| *vsiRNA 4379*(+) | GTTGTTTGCTGGAGACGACATG-Forward | | 22 | | |
| *vsiRNA 4625*(-) | GCAATCTTTGCCTACAGATTA-Forward | | 21 | | |
| *vsiRNA 5839*(-) | TGCTGTTTTCGGGTCGTCTGA-Forward | | 21 | | |
|  | *Pp*-mRNAs Amplified fragment | | | | |
| *PpAGO1* | CTCTGAAAACTCGCTACCAAG-Forward | | | | 153bp |
|  | AACAACACTGGGAAACAATGC-Reverse | | | |
| *PpAGO2* | GGTTTAGGAGGGAGGTTGAGG-Forward | | | | 93bp |
|  | TCAGCCCCATGATGATGTACG- Reverse | | | |
| *PpAGO4* | GAGAAACAAACAAGCGAAGGAAGG-Forward Forward | | | | 114bp |
|  | AGGCACGGTAGATCAGCAGAG- Reverse | | | |
| *PpRDR1* | CCGCCATAGTTGAGTTCACAC- Forward | | | | 103bp |
|  | TTCCTAGCCTTCAGATAAGAGCC- Reverse | | | |
| *PpDCL2* | CCGTTATGTCCCAATCGAGCTG- Forward | | | | 146bp |
|  | CAAGTTCATTCCTCGTGCCAAG- Reverse | | | |
| *PpDCL4* | GCCTGGTCAATTAACGGATT- Forward | | | | 147bp |
|  | TCAATGAAGCCCACATAACT- Reverse | | | |
| *Actin* | CCGGTTCATTACAATTTGACA- Forward | | | | 172bp |
|  | TGACAAGTCGATCCTCCAAA- Reverse | | | |
|  | ASGV mRNA | | | |  |
| *mp* | GCGATTTCCTCATCCGAACTATAC-Forward | | | | 129bp |
|  | ATCTGAAAGTACCAATCCGTCTCC-Reverse | | | |
