# Supplementary material for: Characterization of virus-derived small interfering RNAs in Apple stem grooving virus-infected in vitro-cultured Pyrus pyrifolia shoot tips in response to high temperature treatment
Source: Virol J. 2016 Oct 6;13:166. doi: 10.1186/s12985-016-0625-0 (PMC5053029; doi:10.1186/s12985-016-0625-0)
Supplement: Additional file 5: Table S4. — Pairwise sequence similarities comparing the ASGV-Js2 full-length genome with 17 ASGV isolates sequences from GenBank. (DOC 66 kb) [file 12985_2016_625_MOESM5_ESM.doc]

**Additional file 5:Table S4.** Pairwise sequence similarities comparing the ASGV-Js2 full-length genome with 17 ASGV isolates sequences from GenBank.

| Host | Isolate | Genome length | |  | 5' UTR | |  | ORF1 | | | | |  | ORF2 | | |  | 3'UTR | |
| --- | --- | --- | --- | --- | --- | --- | --- | --- | --- | --- | --- | --- | --- | --- | --- | --- | --- | --- | --- |
|  |  | nt | nt% |  | nt | nt% |  | nt | RdPp | | CP | |  | nt | MP |  |  | nt | nt% |
|  |  |  |  |  |  |  |  |  | nt% | aa% | nt% | aa% |  |  | nt% | aa% |  |  |  |
| Pear | Js2 | 6497 |  |  | 37 |  |  | 6320 |  |  |  |  |  | 963 |  |  |  | 142 |  |
|  | HH | 6496 | 82.8 |  | 36 | 100 |  | 6320 | 81.6 | 96.7 | 94.2 | 98.3 |  | 963 | 85.8 | 96.2 |  | 142 | 95.8 |
|  | SK | 6497 | 80.0 |  | 36 | 97.2 |  | 6320 | 81.5 | 97.6 | 92.7 | 97.0 |  | 963 | 84.3 | 94.4 |  | 143 | 92.3 |
| Apple | P-209 | 6495 | 83.0 |  | 35 | 100 |  | 6320 | 82.4 | 97.6 | 92.1 | 97.0 |  | 963 | 85.6 | 96.6 |  | 142 | 96.5 |
|  | T47 | 6496 | 83.0 |  | 36 | 100 |  | 6320 | 82.3 | 97.6 | 92.3 | 97.0 |  | 963 | 85.4 | 95.9 |  | 142 | 96.5 |
|  | CHN | 6495 | 82.4 |  | 35 | 97.1 |  | 6320 | 82.7 | 98.0 | 93.4 | 97.0 |  | 963 | 84.8 | 94.4 |  | 143 | 93.7 |
|  | YTG | 6495 | 82.9 |  | 35 | 100 |  | 6320 | 80.4 | 97.6 | 94.7 | 98.7 |  | 963 | 85.9 | 97.5 |  | 142 | 96.5 |
|  | AC | 6496 | 82.7 |  | 36 | 100 |  | 6320 | 83.4 | 97.6 | 93.1 | 98.3 |  | 963 | 85.3 | 97.2 |  | 142 | 95.1 |
|  | P12 | 6478 | 82.4 |  | 35 | 100 |  | 6304 | 83.4 | 97.6 | 93.1 | 97.9 |  | 961 | 84.6 | 95.6 |  | 143 | 95.1 |
| Lily | L | 6496 | 82.5 |  | 35 | 100 |  | 6320 | 80.5 | 97.1 | 94.5 | 98.7 |  | 963 | 85.8 | 97.2 |  | 143 | 95.8 |
|  | Li-23 | 6495 | 82.5 |  | 35 | 97.1 |  | 6320 | 81.1 | 97.1 | 94.5 | 98.7 |  | 963 | 86.2 | 97.2 |  | 142 | 95.8 |
| Citrus | PK | 6496 | 81.3 |  | 36 | 97.2 |  | 6320 | 81.0 | 97.1 | 91.3 | 94.1 |  | 963 | 84.6 | 97.2 |  | 142 | 95.1 |
|  | MTH | 6497 | 83.0 |  | 36 | 100 |  | 6320 | 81.8 | 96.7 | 93.2 | 96.6 |  | 963 | 85.7 | 98.4 |  | 143 | 95.8 |
|  | ML | 6495 | 82.5 |  | 36 | 97.2 |  | 6320 | 81.2 | 96.3 | 92.0 | 95.8 |  | 963 | 85.7 | 97.5 |  | 141 | 87.9 |
|  | Lcd-NA-1 | 6496 | 81.5 |  | 35 | 100 |  | 6320 | 82.4 | 97.1 | 91.4 | 97.0 |  | 963 | 84.6 | 96.6 |  | 143 | 95.1 |
|  | STJ | 6497 | 81.2 |  | 36 | 100 |  | 6320 | 81.2 | 97.1 | 91.6 | 95.4 |  | 963 | 83.7 | 96.2 |  | 142 | 93.0 |
|  | K | 6496 | 81.1 |  | 35 | 100 |  | 6320 | 81.0 | 97.1 | 91.7 | 95.8 |  | 963 | 84.2 | 96.9 |  | 143 | 94.4 |
|  | XHC | 6497 | 80.6 |  | 36 | 100 |  | 6320 | 80.8 | 96.7 | 91.0 | 94.9 |  | 963 | 83.0 | 95.9 |  | 143 | 95.8 |
